# “You have to live with some risk, it’s part of the profession”. Specialist ambulance nurses’ perceptions of assignments involving ongoing lethal violence

**DOI:** 10.1186/s13049-023-01082-0

**Published:** 2023-04-05

**Authors:** Susanne Stendahl, Linda Rollgard, Lina Behm, Andreas Rantala

**Affiliations:** 1 Ambulance Service Department, Region Skåne, Ängelholm, Sweden; 2 Ambulance Service Department, Region Skåne, Helsingborg, Sweden; 3 Faculty of Health Science, Kristianstad University, Kristianstad, Sweden; 4 Department of Health Sciences, Lund University, P.O. Box 157, 221 00 Lund, Sweden; 5 Centre of Interprofessional Cooperation Within Emergency Care (CICE), Linnaeus University, Växjö, Sweden

**Keywords:** Ongoing lethal violence, OLV, Ambulance nurse, Prehospital emergency care, Qualitative approach, Phenomenography, Cooperation, Rescue service, Police

## Abstract

**Background:**

As a result of several violent terrorist incidents, authorities in Sweden have shifted from previous approaches of being certain that it is safe for the ambulance service to enter the scene, to a one where “safe enough” is sufficient, potentially making it possible to save more lives. The aim was therefore to describe specialist ambulance nurses' perceptions of the new approach to assignments involving incidents with ongoing lethal violence.

**Methods:**

This interview study employed a descriptive qualitative design with a phenomenographic approach in accordance with Dahlgren and Fallsberg.

**Results:**

Five categories containing conceptual descriptions were developed from the analysis: *Collaboration, Unsafe environments, Resources, Unequipped* and *Risk taking and self-protection*.

**Conclusions:**

The findings highlight the need to ensure that the ambulance service is a learning organisation, where clinicians with experience of an ongoing lethal violence event can pass on and share their knowledge with colleagues to prepare mentally for such an event. Potentially compromised security in the ambulance service when dispatched to ongoing lethal violence incidents needs to be addressed.

## Introduction

Mass Casualty Incidents are events where the number of casualties outnumbers and potentially overwhelms the available resources of e.g., the Emergency Medical Services (EMS) and accident & emergency departments. Such incidents include fires, natural disasters and diseases or pandemics caused by, e.g., influenza, as well as terrorist incidents. Terrorist events or crimes often involve one or two perpetrators, but can also be on a larger number of perpetrators, heavier weapons and more coordinated attacks [1]. Put into perspective, in 2017 there were 10,900 incidents identified as terrorist events worldwide, resulting in 26,400 casualties. As many as 25% of these events occurred in the Middle East, while only two percent took place in Europe and North America. There were 119 terrorist attempts in Europe in 2019, including completed, failed or events that were prevented [2]. Consequently, Swedish authorities have issued a directive on the concept of Ongoing Lethal Violence (OLV), a collaborative working method for the police service, EMS and fire brigade (the so-called blue light authorities) in order to provide the best possible conditions to save lives and contribute to improved health among affected citizens.

An OLV incident is defined as undiscriminating, life-threatening violence directed at a group of people, specific persons, or significant places and/or buildings, lasting until it is interrupted. OLV incidents usually last for no more than 15 min, but it takes time to gain control over the chaos and damage caused [3]. Whether or not an OLV incident should be assessed as terrorism depends on the motive and type of criminal event involved, as the perpetrator(s) often act on an ideological conviction. However, terrorist incidents are not all based on violence, but can take place through threats to harm society at large. Threats and violence are an integral part of terrorist incidents and in some cases they only consist of threats. By using threats, society and states are affected by concerns about the impact on, e.g., the economy and social behaviour, making negotiation or blackmail possible [4]. However, an OLV incident is characterised by its violent substance, not infrequently by means of mass-shootings [5] or bombings where bombs are employed to injure as many as possible and cause chaos [6]. Mass casualty situations call for special measures, e.g., catastrophic haemorrhage control [7] and moving the uninjured to a safe and secluded place. Therefore, preparations to handle the response to a potential OLV incident require interprofessional co-operation and effective communication skills within and between all “blue light authorities” [8]. Familiarisation and mutual understanding have proven to be achieved by joint training sessions and programmes, including table-top experiences, drills and sharing observations and lessons learned [9].

When an OLV incident occurs, the Swedish police service assesses whether it is safe for the staff from blue light authorities to work at the scene, i.e., if the gains outweigh possible risks. In reality, this means that there is a possibility that one or several perpetrators are still on site [3]. Thus, there is a shift from the previous conception of being certain that it is safe for the EMS to enter the scene, to an approach in which *safe enough* is sufficient, potentially making it possible to save more lives. Assignments related to an OLV situation rarely occur, but when they do, the Swedish EMS specialist ambulance nurses’ (SANs) knowledge and skills are in order to save life and alleviate suffering among the injured. Simultaneously, the SANs are expected to work in a potentially unsafe environment where the perpetrator may still be on site or pose a threat from a distance. However, to our knowledge this new way of working has not been reported. The aim was therefore to describe SANs’ perceptions of the new approach to assignments involving incidents with ongoing lethal violence.

## Methods

### Design

In this study a phenomenographic method [10–12] was applied to identify how people understand the world by describing, analysing and interpreting several experiences of a phenomenon. According to Dahlgren and Fallsberg [10], in phenomenography description is superordinate to interpretation, i.e., to arrive at a conceptual description. Thus, the result illustrates similarities and differences in terms of how certain components and aspects are conceived. Originating in educational research [11], the method has been found suitable for identifying concepts in healthcare, as described by Sjöström and Dahlgren [12]. The methodological reporting of this study follows the consolidated criteria for reporting qualitative research (COREQ) standard [13].

### Setting and context

The participants were recruited in a region in southern Sweden with an area of 11,303 km^2^ consisting of 1.4 million inhabitants served by 28 ambulance stations that annually handle some 158, 000 ambulance assignments. The region is divided into four districts, of which two are operated by the county and two by a private contractor on behalf of the region. However, clinical guidelines, quality indicators and staffing requirements are identical, regardless of the mode of operation. Due to national regulations, ambulances in Sweden are crewed by ambulance clinicians of whom at least one is a registered nurse [14]. In the study setting, the registered nurse is required to hold a specialist SAN degree, i.e., 1-year additional university training including a one-year master’s degree in nursing [15]. The other of the two ambulance clinicians could be another SAN, a registered nurse or an emergency medical technician [16].

In recent years EMS organisations, fire brigades and police services have organised realistic joint large scale OLV incident simulations in accordance with guidelines issued by the Swedish Civil Contingencies Agency [3]. In the region where the study was conducted, all operational ambulance clincians participated in both local (i.e., organisations within the primary catchment area) and regional education, training and simulation events.

### Participants

Purposive sampling was chosen to obtain variation in terms of gender, geographical aspects (i.e., both rural and urban settings) and clinical experience, as well as participants with and without experience of an OLV event. However, all participants had participated in at least one large scale OLV simulation event. The inclusion criteria were SANs in the southernmost region in Sweden with a minimum of one year of clinical operative experience. Sixteen SANs (eight women and eight men) participated. They were evenly distributed across the ambulance districts within the county. Their ages ranged from 26 to 48 years (median 41 years) and their clinical experience as SANs ranged from one to 20 years. Half had experienced an event that was characterised as OLV (Table 1).

**Table 1:** Demographics of the participants (N = 16)

	n (%)
Gender	
Men	8 (50)
Women	8 (50)
Age (years)	
< 30	2 (13)
30–40	5 (31)
41–50	7 (43)
51–60	2 (13)
> 60	–
Clinical experience (years)	
1–5	4 (25)
6–10	6 (37)
11–15	2 (13)
16–20	4 (25)
Previously experienced OLV^a^	
Yes	8 (50)
No	8 (50)

^a^Ongoing Lethal Violence

### Data collection

Following approval from the operations manager to conduct the study, the participants were recruited by an independent intermediator. Eligible participants were handed written information about the study including contact information for the first and second authors, who conducted all interviews. The first two authors had previous operational experience from the EMS setting. However, prior to the data collection, their preunderstanding was discussed and reflected on by all the authors in order to become aware of and bridle it. A pilot interview, which was not included in the study, was performed to assess interview techniques and the interview guide. The individual interviews were performed during September 2020 by means of a digital audio/video aid (n = 9), face-to-face (n = 6) and by telephone (n = 1), in accordance with the participants’ wishes. The participants were first asked to describe their operational experience as SANs. The actual interview then began with the main question “When I say ongoing lethal violence, what comes to your mind?” Open-ended follow-up questions were posed to elucidate and deepen the responses. The interviews lasted between 16 and 36 min (median 22 min).

### Analysis

The analysis followed the seven steps described by Dahlgren and Fallsberg [10]. First, the content was discussed by the authors, who read the transcribed material several times (familiarisation). Second, each of the participants’ most significant statements were identified (condensation). Thereafter comparisons were made to find similarities and differences in the material, after which significant statements were grouped and related to each other (grouping). In step five, the categories were formulated and the boundary between different perceptions was decided (articulating). Steps four and five were repeated numerous times to adjust the categories. Next, the categories were named, reflecting the meaning of the data (naming). In the final stage, all categories were assessed by comparing them with each other to confirm that they could not be linked with other categories (contrasting). Quotations were employed to illustrate important results [10].

## Results

**Fig. 1:**
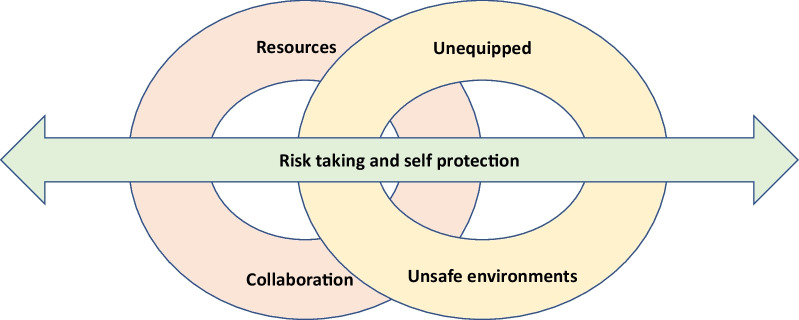
The SANs’ willingness to take risks and adopt self-protection strategies are decisive for how the OLV situation is handled in an unpredictable environment in which they feel unequipped, which is further reinforced by a lack of resources and challenging collaborations

Based on the analysis, there was five categories containing conceptual descriptions representing the SANs’ perceptions of working in situations involving OLV. Each individual interview contained two to five categories (see Table 2). The categories developed were *Collaboration, Unsafe environments, Resources, Unequipped* and *Risk taking and self-protection.* The categories will be presented in more detail below, illustrated by quotations with the participant number in brackets. In addition, they are presented in Fig. 1.

**Table 2:** Individual understanding of the participants’ perceptions of ongoing lethal violence events expressed in the 16 interviews

Interview	1	2	3	4	5	6	7	8	9	10	11	12	13	14	15	16
Collaboration	x	x	x	x	x			x	x	x	x	x	x	x	x	x
Unsafe environments		x	x	x		x	x	x			x	x	x		x	x
Resources	x	x	x		x		x	x	x		x	x	x		x	x
Unequipped	x	x	x	x	x	x	x	x	x	x	x	x	x	x	x	x
Risk taking and self-protection	x	x	x		x		x	x	x	x	x	x	x	x	x	x

### Collaboration

The participants perceived that assignments involving OLV were dependent on collaboration between several organisations. Information passed between the organisations involved was considered a crucial part of collaboration. The participants described a lack of information from the emergency medical dispatch centre when they were assigned to a mission, possibly resulting in vital information that explicitly or implicitly pointed towards a potential OLV incident being left out. Generally, early information from the emergency medical dispatch centre consists of fixed classifications, e.g., "life threatening condition", which can include many possible scenarios in which the patient’s life is in danger, including trauma, medical conditions as well as various types of violence. The SANs believed that the emergency medical dispatch centre prioritises alerting an ambulance before gathering all the necessary information from the caller that could be passed on to the ambulance clinicians. Overall, the participants perceived that emergency medical dispatch centre staff do not possess sufficient knowledge to identify tacit information that could possibly indicate an OLV event and only pass on the information verbally stated by the caller.

Everyday clinical operations sometimes required collaboration with the police service. Even in these usually non-urgent situations, the participants reported difficulties in establishing contact via the designated collaboration radio channel, including inability to obtain feedback from police units who were the first on site. As the police service operated via a separate response channel, the SANs sometimes did not hear important information, which was unintentionally withheld. The perception was that especially in urgent, violent, or threatening situations, the police left the ambulance clinicians to themselves. This created frustration, even among experienced SANs, as without information they could not know what to expect or whether the location was considered sufficiently safe. Problems concerning collaboration were perceived as possibly delaying necessary medical interventions.

It became a..... you got a lump in your stomach, a feeling of insecurity when you were to continue and enter the scene, because you have to take care of your own safety because the police were not on the same designated collaboration radio channel and therefore the communication broke down, so we didn't know what we were getting into (P10)

Furthermore, the SANs stressed that the communication within and between organisations needed to be straightforward and clear, as the internal jargon within the respective organisations could hamper the situation. The SANs pointed out that despite the situation, they trusted police service representatives’ evaluation of whether the place was safe enough to enter.

The participants highlighted the fact that the phenomenon of “borderless routing” [the emergency medical dispatch centre can assign an ambulance to any location within the region] contributed to uncertainty. Ambulance assignments in their own familiar district meant that SANs had knowledge of e.g., residential areas where OLV events were more likely to occur, as well as local knowledge of exit or escape routes and the geography of surrounding areas.

In the past, the SOS [Emergency Medical Dispatch Centre] was tied to a certain catchment area. They had knowledge of the local geographical area. And it's the same with ourselves. Because of the borderless routing we cover a larger geographical area and can end up in places that are dangerous, although we don't know it ourselves. (P2).

It also emerged that the participants perceived operational collaboration and simulation with the local police service and fire brigades as advantageous to prepare for a future OLV incident. The participants also mentioned the need for operational interventions at the scene together with colleagues and organisations from geographical areas other than those they usually work with, as OLV events often result in many injuries and/or causalities.

Say that there is an ongoing shooting at for example the central station. I would not hesitate to team up with the police and enter the site to provide life saving measures here and now. A safe location… yes... what is a safe place? (P8).

Participants who had attended interprofessional simulations of OLV events perceived the ideas and advice provided by police departments as valuable, serving as a kind of mental preparation.

### Unsafe environments

The participants reported that in assignments involving OLV incidents there was a high demand from the public and significant others to simultaneously attend to and save the lives of several severely injured individuals. Furthermore, demands from the public to enter situations or locations that were not considered safe enough were perceived as stressful, especially as the public and significant others generally have no knowledge of EMS working methods. The first ambulance unit at the scene is encumbered by the need to establish an operational management function, making it impossible to provide medical assistance other than trying to obtain a picture of the site and situation. Pressure and sometimes threatening behaviour from the public demand the SANs to save lives despite a lack of safe preconditions were perceived as stressful and challenging. In these situations, the participants requested police presence as a security measure. In addition, the participants strongly objected to being filmed involuntarily and thereafter posted on social media. The role of the media in an OLV situation was perceived as important, but in the short term there were concerns about being identified and possibly publicly questioned.

I feel that the public expect us to handle everything…//When you think about it, what the expectations consist of, it can feel like quite high demands or high expectations, and can we live up to it? Or can I live up to it? (P13).

### Resources

The SANs expressed that they perceived a potential shortage of resources in terms of ambulance availability and personal protection gear in the event of an OLV situation. They pointed out that there is already a shortage of resources in their normal workday, which would become even more severe in the case of an OLV event. Bearing in mind that every minute counts, waiting times for additional ambulances in an OLV event were considered daunting.

It's not always that the Emergency Medical Dispatch Centre can immediately allocate ten ambulances to the scene and for you to establish a staff function, so before the ambulances actually arrive victims may have ended up dead (P11)

Personal protection gear was requested, e.g., protective vests, pepper spray or other types of personal protective equipment. However, it was discussed if wearing protective vests could generate a false sense of safety, possibly leading to exposing oneself to greater dangers than necessary. However, only wearing their ordinary protective clothing, jacket, coveralls and helmet, made the SANs feel unprotected. Furthermore, they pointed out a potential lack of medical materials in the event of OLV. They stressed the limited amount of e.g., tourniquets [stringing in case of limb injury], potentially preventing the ambulance clinicians from taking life-changing measures at an early stage of an OLV event.

Ehhmm so when thinking purely in terms of security, we don't have as much as...that we can protect ourselves with. We have no training in close combat and we have no protective clothing, other than our helmets and...and...so we are quite vulnerable. (P2).

### Unequipped

The SANs perceived that operational experience was of significance in assignments involving OLV. However, because incidents with OLV were rare, the participants considered that they were not properly equipped for working under such conditions. They expressed that medical assessments and triage were performed in everyday clinical operations, but not to the extent necessary for special events such as OLV. The perception among the participants with longer operational experience was that any event that only occurs occasionally leads to a risk of becoming fragmented, forcing experienced ambulance clinicians to handle the event single-handedly, as well as having to supervise less experienced colleague.

The SANs considered that they possess adequate knowledge of how to treat injuries in an OLV event, e.g., controlling catastrophic haemorrhage or airway management with simple aids. However, the perception was that they are not equipped for working in potentially hazardous environments.

Why had no one been able to tell me this before? Is this less important than the fact that the airway should always be managed? I mean, I always know how to take care of the airway in my patient before I do anything else… but if I do not know about my safety or I do not know the situation with shootings... (P10)

The participants perceived a lack of tactical ability to act in OLV incidents and such events place high demands on ambulance clinicians. The SANs expressed a need for local training sessions where they could discuss possible scenarios. They stressed that mental preparation was more important than the practical training, but it would be best if the two could be combined. Like other workplace-based training situations such as cardiopulmonary resuscitation or medical management, the participants requested the preparation of structured action plans for OLV events, as they rarely occur. Structured care was perceived as valuable in such stressful and urgent situations by both experienced ambulance clinicians and new employees.

### Risk taking and self-protection

The participants perceived that they had to act as their own safety advocate, due to lack of a fixed plan for assignments involving OLV incidents. The nature of the incident and the SANs’ willingness to take risks were perceived as decisive for how the situation was handled. The participants described that how the situation was interpreted was determined by any previous experience of OLV, earlier non-OLV professional experiences and the type of risk-taking each individual SAN was willing to be exposed to and act upon. Therefore, according to the participants, the outcome of an OLV event was dependent on the SANs’ assessment. The participants referred to the police service and fire brigade, both of which were perceived to have a fixed structured plan that everyone within the two organisations follows.

So, if you have taken this job, you must consider... that such things can happen and then you have to deal with them. Then you would be super stressed and incredibly... frightened ...for sure (P7).

In the case of an OLV event, the participants mentioned the importance of working with competent and experienced colleagues. Teaming up with colleagues who had several years of professional experience and/or experiences of incidents involving OLV was perceived to provide increased security for the ambulance team. The participants perceived and reflected on the fact that variation in individual risk-taking levels could have had an impact on teamwork at an OLV site due to conflicting views among the members of the ambulance team.

You do not rush into situations in the same way as new colleagues sometimes do, you slow down a bit and stop and think about your own safety in a different way... …you want to come home. (P7)

Consensus prevailed among the participants that personal safety took precedence over caring for patients and that SANs wanted to return home alive. However, the participants stated that risk-taking is part of the profession in certain circumstances.

We must also be able to take some form of risk, so to speak…. in this job it is included. Can I live with that notion? Good! Then I can work in the ambulance service. If I am unable to live with that notion, I am not suitable to for working in the ambulance service. You have to live with some risk. It is part of the profession... (P16).

The SANs faced hazards daily that were considered ordinary clinical events, e.g., traffic accidents, crowded public spaces and the cramped space in the ambulance. They were aware of such risks, realising that they are part of the profession, at least to some extent. Based on their experience, the participants reflected on their own behaviour and caring actions as well as place and space, which can be partially implemented in an OLV event.

## Discussion

This study reports SANs’ perceptions of the new approach for assignments involving OLV. In recent years such events have received attention from the public, media and politicians, while the workspace in an OLV event has become more unsafe for SANs as the scene no longer has to be completely safe, merely safe enough to work in.

Our findings indicate that SANs expressed concerns about the lack of operational tactical concepts in the studied region, thus highlighting the lack of security for ambulance clinicians. An ordinary day in an ambulance was described as hazardous by the participants, who accepted risks as part of the profession. Similarly, violence has been described as common in EMS settings in other countries [17]. However, our results show that despite the constant exposure to violent settings, the OLV event was described as something bigger that the participants did not feel fully equipped for, regardless of their training to be prepared for the unexpected [18]. The nature of an OLV event was perceived as generating unpredictability that was present throughout the entire chain of events, i.e., from dispatch until the mission was completed. Thus, the participants stressed that they feel well equipped for the treatment of the patient for which they were trained, but not for doing so in a hazardous environment. The SANs highlighted the need for both physical and mental preparation in order to carry out their assignments. Current studies on OLV events often focus on mass shootings [19], stating that one strategy to educate and prepare the staff is to simulate the event. One simulation of a mass shooting in a hospital in the USA was found to have increased staff knowledge and preparedness by as much as 70% [20]. Furthermore, it is recommended that such simulation exercises should include both practice and reflective conversations [21]. The participants in the present study suggested interprofessional simulations and those who had experienced such simulations stated that the ideas and advice they picked up from, for example, the police service, served as mental preparation. Another strategy used by the participants to feel more secure was to work with a competent and experienced colleague, which has been found to be crucial when exposed to complex situations [22, 23]. The feeling of security increased if colleagues had several years of professional experience and/or experiences of incidents involving OLV, which in previous research has been described as relying on past experiences, and tends to facilitate situational awareness and decision-making [24].

Apart from feeling unequipped and lacking a fixed plan, potentially affecting personal safety and patient care, the participants stressed both internal and external pressures. In the case of an OLV event, internal pressure involve the participants being concerned about the resources at their disposal as they were already perceived as lacking in their daily work. They considered that there could be a potential shortage of ambulances, staff and medical supplies at the scene. Studies have shown that one advantage of simulation exercises is that gaps or limitations in plans, protocols or procedures are identified, enabling solutions to be implemented [20, 21]. SANs also drew attention to external pressure from the public, consisting of expectations to enter into situations that the SANs considered not safe enough. This phenomenon has previously been described by SANs [25, 26] as resulting in pressure to fulfil expectations that cannot be met. In agreement with previous studies [25, 26], the results showed that SANs perceived that the public has no knowledge of EMS working methods. Ahl and Nyström [25] suggest general information to the public about the role of pre-hospital care in order to create realistic expectations, thereby alleviating stress for ambulance clinicians.

Preparing ambulance clinicians for an OLV event is crucial. Existing knowledge indicates that being exposed to or feeling unprepared for violent situations can contribute to stress, which may result in post-traumatic stress disorder and depression [27], as well as feelings such as fear, anxiety, exhaustion, powerlessness and uncertainty [28, 29]. Exposure to violence has been identified as a reason why ambulance clinicians have a higher rate of burn-out than other healthcare professionals [30, 31]. A core perception was that to some extent SANs are prepared to accept the fact that their work is associated with certain risks, although some participants stated that they did not agree to the new approach of “safe enough” when they commenced employment with the EMS, meaning that the organisation has not taken their situation into account.

### Limitations

In terms of methodological quality, this study adheres to the four quality criteria for ensuring trustworthiness in qualitative research developed by Lincoln and Guba [32]. The relatively short duration of the interviews could possibly pose a threat to credibility. However, the participants were very focused on the phenomenon of interest, as they had been informed of the study’s aim in advance, in combination with their major interest in the subject. In addition, the data collected were assessed as rich and contained many different perceptions. The participants were interviewed in a location of their choice, resulting in data collection by means of digital aids (n = 9), face-to-face interviews (n = 6) and by telephone (n = 1). Being unable to collect data face-to-face, i.e., in this case primarily by telephone and to some extent digitally, eliminates non-verbal communication, which must be considered. Dependability may have been influenced by the fact that eligible participants were approached by an independent intermediator, although the varied and qualitatively different perceptions indicate that this did not represent a limitation. Confirmability was ensured as the participants described perceptions of the assignments involving incidents with OLV, which corresponded to the aim of the study and provided rich data. The clinical pre-understanding of three of the authors gained from working as ambulance clinicians in the EMS for many years could potentially have had an influence on the understanding and analysis. However, they did not work together with any of the study participants. Furthermore, there was an open dialogue with the third author who has no clinical experience of this context but extensive knowledge of qualitative research, which helped reduce potential misinterpretation and ensured the confirmability and trustworthiness of the study. Finally, transferability can be deemed high as the participants embody catchment areas comprising both rural and urban settings, as well as diversity in terms of socio-economic status. However, perceptions of assignments involving OLV events could depend on factors such as the workplace culture and management, thus limiting transferability to contexts that differ from the specific EMS context in the study.

## Conclusion

The results of this study indicate that the participants did not feel equipped to work in potentially hazardous environments, lacking educational sessions, reflections as well as real life simulations. Furthermore, the participants perceived the organisation as lacking a fixed plan of action, possibly creating uncertainty and a need to take care of one’s own safety. Thus, it is vital to address the challenges of potentially compromised security in the EMS organisation when dispatched to OLV incidents. We argue that the topic should also be addressed at the political and organisational level.

The findings highlight the need to ensure that the EMS is a learning organisation, where clinicians with experience of an OLV event can pass on and share their knowledge with colleagues as part of creating a mental preparation for an OLV event Moreover, there should be emphasis on the need for regular simulations in collaboration with “blue light organisations”.

## Data Availability

The datasets generated and/or analysed during the present study are not publicly available due to participants’ confidentiality but are available from the corresponding author on reasonable request.

## Abbreviations

EMS: Emergency Medical Services
OLV: Ongoing Lethal Violence
SAN: Specialist Ambulance Nurse
